# Critical Care Ultrasound-Guided Targeted Volume Management as Precision Medicine: Predicting Clinical and Financial Impacts

**DOI:** 10.3390/jpm16100493

**Published:** 2026-09-24

**Authors:** Carolyn S. Kaufman

**Affiliations:** Department of Medicine, Stanford University School of Medicine, Palo Alto, 94305 CA, USA; carolynstarkkaufman@gmail.com

**Keywords:** critical care ultrasound, targeted volume management, fluid responsiveness, fluid tolerance, health economics, return on investment

## Abstract

Precision medicine in the intensive care unit (ICU) is increasingly defined by individualized, bedside physiologic assessment rather than protocolized treatment algorithms. Critical care ultrasound for targeted volume management (CCUS-TVM) operationalizes this shift by helping clinicians answer two questions before administering intravenous (IV) fluids: is it *safe*, and will it be *effective*? Static measures, such as inferior vena cava (IVC) assessment and the Venous Excess Ultrasound Score (VExUS), identify venous congestion and predict fluid *intolerance*, while dynamic measures, such as change in left ventricular outflow tract velocity time integral or carotid flow time after passive leg raise, predict volume *responsiveness*. Together, these tools allow clinicians to tailor resuscitation to an individual patient’s underlying hemodynamics, complementing the four-interface model of shock physiology. Recent meta-analyses and the 2024 Society and Critical Care Medicine and 2026 Surviving Sepsis Campaign guidelines show that CCUS-TVM—particularly dynamic assessment—contributes to fewer episodes of renal replacement therapy and mechanical ventilation, shorter ICU stays, and decreased mortality. Beyond clinical benefit, this communication presents an original, exploratory estimate of the potential per-patient cost savings associated with dynamic CCUS-TVM in sepsis— approximately $2600–4400—driven by reductions in renal replacement therapy, mechanical ventilation, and ICU length of stay. Taken together, CCUS-TVM offers a clinically validated and economically favorable framework for advancing precision medicine in resuscitation.

## 1. Introduction

Patients with organ hypoperfusion require rapid bedside evaluation and clinical decision-making that can be significantly improved through physiologic assessments with point-of-care ultrasound (POCUS) [1]. When faced with a patient suffering from end-organ hypoperfusion, the clinician has essentially two options for acute intervention: administration of intravenous (IV) fluids or administration of vasoactive medication [2]. Historically, the default option has been reflexive IV fluid administration, often in large amounts (for example, 30 mL/kg, which equates to almost half the average patient’s total blood volume) [3,4,5]. However, clinicians increasingly recognize potential harm from excessive IV fluid administration, and critical care leaders now recommend a more nuanced approach to targeted volume management, one that specifically utilizes ultrasound to guide resuscitation [6]. This brief correspondence is an overview of the clinical and financial benefits of implementing POCUS to guide tailored IV fluid resuscitation.

### 1.1. CCUS-TVM for Precision Medicine in Resuscitation

When a clinical indication for IV fluid is established, i.e., clinical hypoperfusion as described above, critical care ultrasound for targeted volume management (CCUS-TVM) allows for precision medicine by tailoring resuscitation to an individual patient’s unique pathophysiology. Fundamentally, CCUS-TVM helps answer two mutually exclusive, but critically important questions: (1) is it *safe* to give IV fluid? and (2) will IV fluids be *effective*? Specifically, bedside ultrasound can uncover evidence that IV fluid may not be *safe* for a particular patient (e.g., if there are extensive B-lines on lung ultrasound, the inferior vena cava (IVC) appears enlarged/non-collapsible, or the jugular Doppler venous waveform suggests congestion) [7,8]. More involved bedside techniques such as the Venous Excess Ultrasound Score (VExUS) can provide evidence of congestion in the systemic circulation, which would also predict intolerance to IV fluids [9]. These static measures can predict downstream harm to organs susceptible to damage from volume overload (e.g., kidneys, lungs) if the patient receives IV fluids. For example, one prospective cohort study of 138 general intensive care unit (ICU) patients showed a VExUS score of 2 or greater—indicating venous congestion at baseline—was independently associated with major adverse kidney events at 30 days [10]. However, this association may be less consistent in sepsis-specific populations, as a separate multicenter prospective study of 108 septic patients found no significant relationship between VExUS 2 or greater and acute kidney injury, 30-day mortality, or receipt of renal replacement therapy [11]. This suggests that prognostic performance may vary by patient population or cohort. Additionally, fluid tolerance itself is not a single binary construct. One recent epidemiologic analysis combining lung ultrasound with VExUS found that isolated pulmonary congestion (defined by B-lines on lung ultrasound), isolated systemic congestion (defined by elevated VExUS score), and combined pulmonary-systemic congestion occur at meaningfully different frequencies among critically ill patients [12], indicating that these represent partially distinct physiologic processes rather than a single wet or dry dichotomy. By utilizing multiple static ultrasound methods to evaluate for volume overload, clinicians can tailor resuscitation efforts to the individual’s unique physiology, facilitating precision medicine which can improve patient outcomes. Fundamentally, the question of fluid safety is synonymous with fluid tolerance [13,14], and a patient can be reasonably categorized as likely fluid intolerant if they demonstrate signs of volume overload at baseline.

Importantly, static measures such as IVC ultrasound, lung ultrasound, and VExUS may signal for or against *safety* of IV fluid administration but do not predict *efficacy* of IV fluid administration [13]. In fact, many studies show no correlation between IVC size/diameter and volume responsiveness [15,16]. Instead, clinicians can utilize dynamic measures of cardiac output and stroke volume to measure change after preload challenge, which better predicts whether the patient will be volume responsive in addition to volume tolerant [17]. For example, clinicians can record change in stroke volume surrogates such as left ventricular outflow tract velocity time integral (LVOT-VTI) or corrected carotid flow time (ccFT) after a passive leg raise to predict whether the individual patient will benefit from increased stroke volume (and presumably improved cardiac output and subsequent end-organ perfusion) if IV fluid is administered [18,19]. If CCUS-TVM signals a patient will both tolerate and benefit from IV fluid, the clinician can feel much more confident about resuscitating with volume prior to reaching for vasoactive medication. Alternatively, if ultrasound findings suggest IV fluid is unsafe and/or ineffective for the patient, clinicians may opt for the safer and more effective method (e.g., vasoactive medication like norepinephrine) at an earlier timepoint, which may prevent downstream complications of volume overload [13]. One recent multicenter observational study explored this relationship between fluid safety and efficacy in critically ill patients [20]. A fluid challenge significantly worsened venous congestion (measured by VExUS) only in patients classified as fluid non-responsive (defined by a cardiac index increase of less than 15% after a 500 mL fluid challenge) but not in responsive patients [20], suggesting patients who do not augment cardiac index after a fluid bolus are significantly more likely to experience volume overload if resuscitated with IV fluid. These physiologic assessments complement the emerging four-interface model of hemodynamics by identifying abnormalities in ventricular–arterial coupling and systemic venous congestion that contribute to impaired organ perfusion [21].

### 1.2. CCUS-TVM Clinical Impact

With many new methods developed for CCUS-TVM, there is increasing interest in understanding the impact of these precision medicine interventions both clinically for the patient and financially for the institution. One recent meta-analysis of randomized controlled trials found that ultrasound-guided fluid resuscitation in patients reduced mortality compared to standard care [22]. Patients treated with CCUS-TVM were found to have significantly lower 24 h fluid balance and significantly increased vasoactive medication use, suggesting the mortality benefit may be related to earlier employment of vasoactive medications when CCUS-TVM signaled lack of safety and/or efficacy for IV fluids [22]. These benefits were found to be most strongly related to interventions involving evaluation of the inferior vena cava and ultrasound measures of change with dynamic testing such as passive leg raise [22]. Another recent meta-analysis including a broader population of acutely ill patients found similar effects; patients randomized to receive CCUS-TVM had reduced all-cause mortality, potentially due to significantly lower 72 h fluid balance [23]. Overall, evidence increasingly suggests improved outcomes when patients are treated with precision medicine via CCUS-TVM techniques [22,23,24]. Acknowledging this rapid evolution of critical care ultrasound, the Society of Critical Care Medicine released updated guidelines in 2024 to specifically advocate for the use of CCUS-TVM to guide resuscitation in all acutely ill patients [6].

Dynamic CCUS-TVM assessments may be even more beneficial for patient outcomes than static assessments [24,25]. Static CCUS-TVM includes measures obtained at baseline such as inferior vena cava size and collapsibility [15,16]. Dynamic CCUS-TVM refers to measuring an acute change in physiologic variable(s) after an intervention—for example, assessing change in stroke volume after performing passive leg raise to increase venous return and therefore cardiac preload [17,18,19]. One recent meta-analysis that evaluated the use of dynamic measures of fluid responsiveness to guide resuscitation compared with other methods for patients with sepsis and septic shock found significantly reduced risk of acute kidney injury and mortality, with an estimated 103 lives saved per 1000 patients treated with dynamic assessments [24]. In fact, accumulating evidence for the clinical benefit of dynamic assessments resulted in the most recent Surviving Sepsis Campaign Guidelines formally recommending “using dynamic measures to guide fluid resuscitation over physical examination or static measures alone” [25]. As a form of precision medicine, targeting bedside physiology with CCUS should emphasize dynamic assessments in addition to static measures of patient-specific physiology.

### 1.3. CCUS-TVM Financial Impact

In addition to improved patient outcomes, there may be financial benefits at the institution level when employing CCUS-TVM for precision in resuscitation. For example, sepsis is the leading cause of hospitalization and mortality in the United States, representing a primary driver of acute care expenditures [26,27,28]. As of 2026, the Centers for Medicare & Medicaid Services (CMS) have tied a portion of hospital reimbursement to the SEP-1 quality bundle, which requires documentation of repeat volume status and tissue perfusion assessment following initial fluid resuscitation in patients with septic shock [29]. Health systems that fail to provide objective evidence of hemodynamic status face financial penalties under Value-Based Purchasing (VBP) programs [30]. Consequently, the adoption of dynamic CCUS-TVM assessments is not merely a clinical preference but a strategic response to a shifting regulatory and financing landscape. Hospitals can avoid reimbursement penalties if clinicians document CCUS-TVM findings to justify a change in target bolus volume (e.g., “30 mL/kg was not administered due to ultrasound evidence of fluid intolerance or inefficacy”). Additionally, repeat ultrasound assessment can fulfil the required 6 h perfusion/shock reassessment. Through documentation of CCUS-TVM in these instances, institutions can ensure reimbursement for resource-intensive care of the septic patient while also improving clinical outcomes [25,29,30].

Estimating Return on Investment (ROI) for CCUS-TVM is complex but relevant to the institution. Sepsis is a particularly salient example due to not only the prevalence of the diagnosis but also the above-mentioned need for targeted resuscitation. From a health economics perspective, sepsis management is largely governed by Prospective Payment Systems (PPS), through which hospitals are reimbursed via fixed Diagnostic Related Group (DRG) bundles [31]. Under this financing structure, the hospital acts as a residual claimant; any costs incurred beyond the fixed reimbursement—specifically “absorbed” variable costs such as prolonged mechanical ventilation, renal replacement therapy, and extended ICU stays—directly reduce the institution’s net margin [32,33]. Therefore, reducing the “absorbed” variable costs directly increases the ROI for each patient case.

The 2026 Surviving Sepsis Campaign Guidelines conducted an independent meta-analysis to estimate the impact of utilizing dynamic assessments (such as change in stroke volume after passive leg raise) to guide fluid resuscitation for septic patients [25]. This expert panel estimated that dynamic assessments prevent 71 episodes of renal replacement therapy (RRT) per 1000 patients treated [25]. RRT carries an estimated cost of $919 per day [34] and has a median duration of 6 days of treatment [35], bringing the total estimated cost to around $5500 per RRT episode. Considering the number needed to treat of 14 patients [25], this equates to an estimated savings of around $400 per patient. There are further financial savings when considering reduction in mechanical ventilation (MV) days. The 2026 Surviving Sepsis Campaign Guidelines estimated an average of 0.79 ventilator days prevented per patient who underwent dynamic assessments to guide resuscitation [25]; using an estimated cost of MV of $2800 per patient-day [36], this equates to around $2200 saved per patient. In combination, the utilization of dynamic assessments to guide fluid resuscitation in septic patients may save an estimated $2600 per patient based on the outcomes predicted by the 2026 Surviving Sepsis Campaign independent meta-analysis [25]. Notably, these guidelines did not comment on the expected impact of dynamic assessments on ICU length of stay. However, a recent meta-analysis estimated an average reduction of 0.38 ICU days per septic patient resuscitated using dynamic assessments compared to static or other measures [24]; using an estimated cost of $6000 per ICU bed day minus the estimated cost of $1300 per floor bed day for a $4700 net difference [37], this equates to around $1800 saved per patient. Combined with the predicted savings from preventing RRT and MV events, this suggests an estimated savings of $4400 per septic patient treated with dynamic assessments. It is important to emphasize that ICU length of stay, mechanical ventilation duration, and RRT requirement are not fully independent cost domains in critically ill patients. For example, a shorter ICU stay may itself partly reflect earlier liberation from mechanical ventilation or avoidance of RRT. These three estimates should therefore not be interpreted as strictly additive; the combined $4400 figure is presented as an approximate, upper-bound illustration of potential savings rather than a precise, non-overlapping sum. ROI is conventionally calculated as the difference between the cost savings and cost of the intervention divided by the cost of the intervention. As this commentary does not model the actual costs of CCUS-TVM implementation, a formal ROI cannot be calculated here. Instead, these modeled savings are best interpreted as defining a break-even threshold: an intervention costing less than approximately $2600–4400 per patient could plausibly generate net cost savings for the institution. Taken together, dynamic CCUS-TVM to guide resuscitation may benefit institutions financially in addition to improving patient outcomes.

## 2. Limitations

This brief correspondence has several limitations. First, the clinical conclusions are based on published randomized trials, meta-analyses, and guideline recommendations, and therefore remain subject to the heterogeneity and methodological limitations of the underlying studies. Second, the financial analysis is intended to illustrate the potential economic impact of critical care ultrasound for targeted volume management (CCUS-TVM) rather than represent a formal cost-effectiveness analysis. Estimated cost savings are based on published reductions in resource utilization and representative hospital cost estimates adjusted for inflation and may not be generalizable across institutions with differing reimbursement models, implementation costs, or practice patterns. Finally, successful implementation of CCUS-TVM depends on clinician training and workflow integration, all of which may influence clinical outcomes and financial return. Prospective implementation and cost-effectiveness studies are needed to validate these findings across diverse healthcare settings.

A limitation of this simple health economic analysis is that there are overlapping cost variables in the ICU (e.g., length of stay, mechanical ventilation and renal replacement therapy) that may not sum directly. In addition, the meta-analyses included consider both patients with septic shock and mixed populations of acutely ill patients. Though true, a recent database study consisting of approximately 34,000 critically ill patients on mechanical ventilation employed an elegant mathematical model to predict the ‘expected’ amount of IV fluid that was risk-adjusted for patient comorbidities and compared that to the ‘observed’ IV fluid received by the patients [38]. The authors performed a detailed cost analysis and found that when the observed IV fluid received was greater than the model-expected amount—by as little as 1.5 L—the cost of care was approximately $4300 USD (2026 adjusted) more for the patients who received greater than the expected amount of IV fluid. This dollar amount attributed to excessive IV fluid is comparable to the number presented herein despite different methods of calculation. This increases the external validity of the economic model despite its methodological shortcomings.

## 3. Conclusions

Precision medicine in critical care is increasingly defined by individualized physiologic assessment rather than protocolized treatment algorithms. Recent Society of Critical Care Medicine guidelines advocate for the use of ultrasound during resuscitation—termed critical care ultrasound for targeted volume management (CCUS-TVM)—for all acutely ill patients [6]. CCUS-TVM transforms resuscitation from protocolized fluid administration toward continuous, patient-specific management guided by dynamic cardiovascular physiology. Dynamic assessments may be more beneficial than static measures of patient physiology when predicting fluid responsiveness [17,19,20]. Multiple studies have demonstrated improved patient outcomes when utilizing CCUS-TVM to predict the safety/efficacy of IV fluid to guide resuscitation efforts, and CCUS-TVM may offer institutions potential cost savings, warranting further prospective studies [6,18,19,20]. Especially in sepsis and septic shock, the combination of clinical benefits and economic feasibility supports wider adoption of CCUS-TVM to enhance precision medicine in resuscitation.

## Data Availability

No new data were created or analyzed in this study. Data sharing is not applicable to this article.

## List of Abbreviations

VExUS	Venous excess ultrasound score
LVOT VTI	Left ventricular outflow tract velocity time integral
IV	Intravenous
IVC	Inferior vena cava
CCUS	Critical care ultrasound
TVM	Targeted volume management
PLR	Passive leg raise
RRT	Renal replacement therapy
MV	Mechanical ventilation
